# 2,2′-{[2-(2-Hy­droxy­phen­yl)-4-methyl­imidazolidine-1,3-di­yl]bis­(methyl­ene)}diphenol

**DOI:** 10.1107/S1600536813019417

**Published:** 2013-07-20

**Authors:** Augusto Rivera, Lorena Cárdenas, Jaime Ríos-Motta, Monika Kučeráková, Michal Dušek

**Affiliations:** a Departamento de Química, Facultad de Ciencias, Universidad Nacional de Colombia, Sede Bogotá, Cra 30 No. 45-03, Bogotá, Código Postal 111321, Colombia; b Institute of Physics, Academy of Sciences of the Czech Republic v.v.i., Na Slovance 2, 182 21 Praha 8, Czech Republic

## Abstract

The asymmetric unit in the title compound, C_24_H_26_N_2_O_3_, comprises two independent mol­ecules (*A* and *B*). In molecule *A*, the central 2-hydroxyphenyl ring is inclined to the mean plane of the major component of the imidazolidine ring by 84.52 (14)°, and by 68.08 (9) and 47.48 (9)° to the outer phenol rings. The later are inclined to one another by 66.76 (9)° and by 78.12 (14) and 80.20 (14)° to the imidazoline ring mean plane. In molecule *B*, the central 2-hydroxyphenyl ring is inclined to the mean plane of the imidazolidine ring by 73.64 (10)°, and by 75.60 (8) and 38.32 (9)° to the outer phenol rings. The later are inclined to one another by 69.47 (9)° and by 82.60 (10) and 64.26 (10)° to the imidazolidine ring mean plane. In each of the independent mol­ecules, two intra­molecular O—H⋯N hydrogen bond form *S*(6) ring motifs. In disordered mol­ecule *A*, the O—H groups of the 2-hy­droxy­benzyl groups are also involved in intra­molecular O—H⋯O hydrogen bonds, with the O atom of the hy­droxy­phenyl group acting as the acceptor. In the crystal, *A* molecules are linked by pairs of O—H⋯O hydrogen bonds forming inversion dimers. These dimers are linked to the *B* molecules *via* O—H⋯O hydrogen bonds forming double-layered slabs lying parallel to the *bc* plane.

## Related literature

For related structures, see: Rivera *et al.* (2012 ▶, 2013*b* ▶,*c* ▶). For the synthesis of the title compound, see: Rivera *et al.* (2013*a* ▶). For reference bond-length data, see: Allen *et al.* (1987 ▶). For hydrogen-bond graph-set nomenclature, see: Bernstein *et al.* (1995 ▶). For Cremer–Pople ring-puckering parameters, see: Cremer & Pople (1975 ▶).
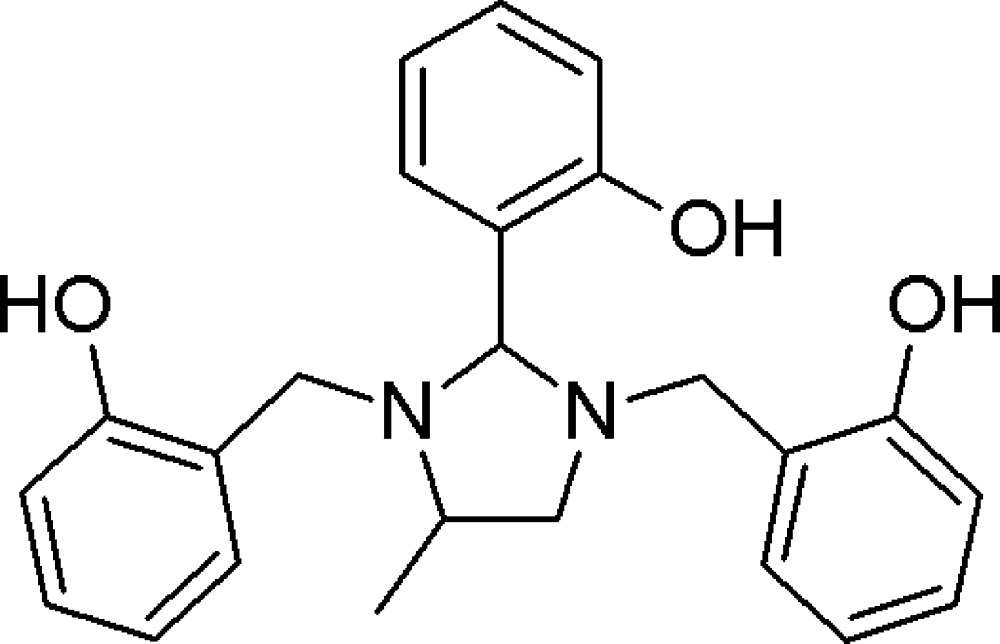


## Experimental

### 

#### Crystal data

C_24_H_26_N_2_O_3_
*M* *_r_* = 390.47
Monoclinic, 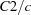

*a* = 24.2482 (10) Å
*b* = 9.8145 (3) Å
*c* = 35.1675 (15) Å
β = 102.450 (4)°
*V* = 8172.5 (6) Å^3^
*Z* = 16
Cu *K*α radiation
μ = 0.67 mm^−1^
*T* = 120 K
0.20 × 0.11 × 0.05 mm

#### Data collection

Agilent Xcalibur Gemini ultra diffractometer with Atlas detector
Absorption correction: multi-scan (*CrysAlis PRO*; Agilent, 2010 ▶) *T*_min_ = 0.505, *T*_max_ = 1.000
15999 measured reflections
7053 independent reflections
5315 reflections with *I* > 3σ(*I*)
*R*_int_ = 0.027

#### Refinement

*R*[*F* ^2^ > 2σ(*F* ^2^)] = 0.040
*wR*(*F* ^2^) = 0.048
*S* = 1.68
7053 reflections
556 parameters
7 restraints
H atoms treated by a mixture of independent and constrained refinement
Δρ_max_ = 0.31 e Å^−3^
Δρ_min_ = −0.19 e Å^−3^

### 

Data collection: *CrysAlis PRO* (Agilent, 2010 ▶); cell refinement: *CrysAlis PRO*; data reduction: *CrysAlis PRO*; program(s) used to solve structure: *SUPERFLIP* (Palatinus & Chapuis, 2007 ▶); program(s) used to refine structure: *JANA2006* (Petříček *et al.*, 2006 ▶); molecular graphics: *DIAMOND* (Brandenburg & Putz, 2005 ▶); software used to prepare material for publication: *JANA2006*.

## Supplementary Material

Crystal structure: contains datablock(s) global, I. DOI: 10.1107/S1600536813019417/sj5345sup1.cif

Structure factors: contains datablock(s) I. DOI: 10.1107/S1600536813019417/sj5345Isup2.hkl

Supplementary material file. DOI: 10.1107/S1600536813019417/sj5345Isup3.cml

Additional supplementary materials:  crystallographic information; 3D view; checkCIF report

## Figures and Tables

**Table 1:** Hydrogen-bond geometry (Å, °)

*D*—H⋯*A*	*D*—H	H⋯*A*	*D*⋯*A*	*D*—H⋯*A*
O1—H1⋯N3	0.883 (14)	1.838 (16)	2.6344 (17)	148.9 (19)
O2—H2⋯N4	0.883 (15)	1.937 (16)	2.7360 (17)	149.7 (18)
O3—H3⋯N2	0.883 (16)	1.839 (17)	2.6680 (17)	155.5 (19)
O4—H4⋯N1	0.883 (19)	1.849 (19)	2.646 (2)	149.0 (14)
O1—H1⋯O5	0.883 (14)	2.472 (19)	3.0381 (18)	122.4 (16)
O4—H4⋯O5	0.883 (14)	2.523 (17)	3.1167 (15)	125.3 (15)
O5—H5⋯O1^i^	0.883 (14)	1.930 (15)	2.8108 (18)	174.9 (16)
O6—H6⋯O4^ii^	0.883 (16)	1.907 (14)	2.746 (2)	157.9 (18)

Symmetry codes: (i) 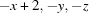; (ii) 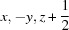.

**Fig. 1.**
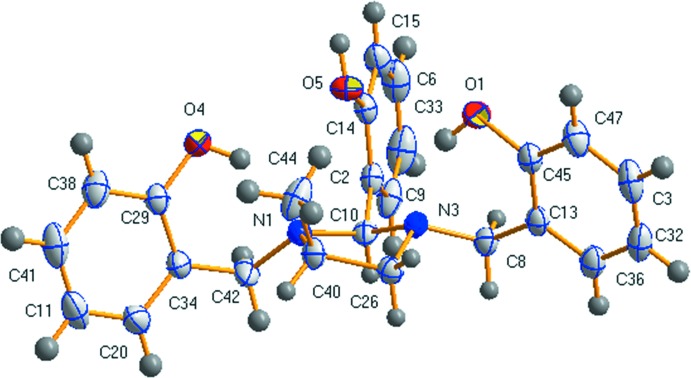
A perspective view of the molecule A of the title compound. Displacement ellipsoids are drawn at the 50% probability level and H atoms are shown as small spheres of arbitrary radii. Hydrogen bonds are drawn as dashed lines. For clarity, only the major disorder component of the disordered C26, C40, C44 segment of the imidazolidine ring is shown.

**Fig. 2.**
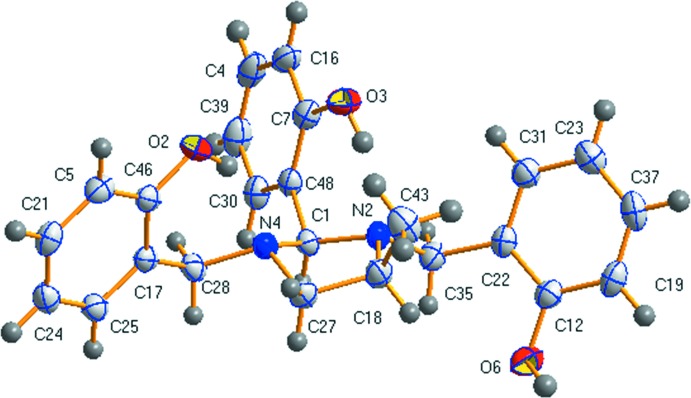
A perspective view of the molecule B of the title compound. Displacement ellipsoids are drawn at the 50% probability level and H atoms are shown as small spheres of arbitrary radii.

## supplementary crystallographic information

### Comment

The asymmetric unit of the title compound, C_48_H_52_N_4_O_6_, contains two independent molecules, Figs. 1 and 2. The C26 and C40 atoms from the imidazolidine ring of molecule A together with the C44 methyl substituent and their H atoms were disordered over two positions with relative occupancies 0.7543 (7):0.2456 (3). The second molecule (B, Fig. 2) appeared to exhibit a similar disorder, but the relative occupancies of the major component of C43 refined to approximately 0.92 and the two additional C atoms of the minor disordered chain could not be localized in difference Fourier map. Therefore, we did not introduce disorder into the structural model of the second molecule. The imidazolidine rings in both molecules adopt envelope conformations, as indicated by the ring-puckering parameters, Q(2) = 0.4293 (18) Å, φ(2) = 245.1 (2)° envelope on N4, and Q(2) = 0.433 (4) Å, φ(2) = 78.6 (4)° envelope on N3 (Cremer & Pople 1975). In molecule A, the phenol rings form a dihedral angle of 67.25 (18)°, whereas the corresponding angle is 69.63 (13)° in the other. The 2-hydroxybenzyl ring forms dihedral angles of 79.55 (15) and 70.74 (15)° with the mean plane of the imidazolidine ring in molecule A, and 56.52 (17) and 75.51 (13)° in molecule B. The dihedral angles between the latter planes and the adjacent phenyl rings are 76.39 (17)° and 57.92 (17)°. All bond lengths (Allen *et al.*,1987) are normal and correspond to those observed in related compounds (Rivera *et al.*, 2012, 2013*b*,*c*).

In molecule A, the O1 and O4 *o*-hydroxybenzyl groups act as donors, forming S(6) hydrogen-bond motifs with the N3 and N1 nitrogen atoms (Bernstein *et al.* 1995), while in molecule B, the hydrogen-bond donors are atoms O3 and O2, Table 1. In the disordered molecule, the O—H groups of the 2-hydroxybenzyl groups are also involved in weaker intramolecular O—H···O hydrogen bonds, with the O5 atom acting as the acceptor. The N··· O distances show that the strongest intramolecular hydrogen bond is formed in the case of O1 and the weakest one for O2. But this value, 2.7360 (17), is still shorter than those observed in related structures [2.7569 (18) and 2.721 (3) Å] (Rivera *et al.*, 2012, 2013*b*). However, the observed N4···O2 bond distance is slightly longer compared to the mean value [2.722 (3) Å] observed in the *p*-chloro derivative (Rivera *et al.*, 2013*c*). Intermolecular O5—H5···O1 and O6—H6···O4 hydrogen bonds also consolidate molecules in the crystal structure, Table 1.

### Experimental

Following our former methodology Rivera *et al.* (2013*a*), salicylaldehyde was added to a stirred toluene solution of 2,2'-[propane-1,2-diylbis(iminomethanediyl)]diphenol in a molar ratio of 1.0:1.1. The resulting mixture was heated under reflux for 12 h. After cooling to room temperature, the solvent was evaporated on a rotary evaporator, the residue was cooled, and the precipitate was filtered off, washed with cold ethanol, dried in air, and recrystallized from ethanol, yielding colourless crystalline flakes, mp 167–168 °C.

### Refinement

The imidazolidine ring was found to be disordered over two positions, with relative occupancies 0.7543 (7):0.2456 (3). The C atoms of the both disorder components (minor and major) were refined harmonically with the ADP of C40' equal to that of C40 and that of C26' equal to ADP C26, due to the close proximity of these atoms. ADP values of the terminal C44 and C44' methyl groups were refined independently. The distances C40—C26 and C40—C44 were kept equal to those of C40'—C26' and C40'—C44'. The occupancies of the minor and major components refined so as to sum to unity.

The second molecule (B) exhibits a similar kind of disorder, but the relative occupancies of the methyl carbon refined to approximately 0.92 while the other methyl carbon occupancy was 0.08. Due to the very low occupancy of the minor component, we did not introduce disorder of the second molecule into the final structure model.

The hydroxyl H atoms were found in difference Fourier maps and their coordinates were refined with a distance restraint d(O—H) = 0.878 Å with σ 0.01. All other H atoms were kept in the geometrically correct positions with C—H = 0.96 A. The isotropic atomic displacement parameters of H atoms were evaluated as 1.2*U*_eq_ of the parent atom.

### Crystal data

**Table:**

C_24_H_26_N_2_O_3_	*F*(000) = 3328
*M**_r_* = 390.47	*D*_x_ = 1.269 Mg m^−^^3^
Monoclinic, *C*2/*c*	Cu *K*α radiation, λ = 1.5418 Å
Hall symbol: -C 2yc	Cell parameters from 7208 reflections
*a* = 24.2482 (10) Å	θ = 3.7–67.0°
*b* = 9.8145 (3) Å	µ = 0.67 mm^−^^1^
*c* = 35.1675 (15) Å	*T* = 120 K
β = 102.450 (4)°	Polygon, colourless
*V* = 8172.5 (6) Å^3^	0.20 × 0.11 × 0.05 mm
*Z* = 16	

### Data collection

**Table:**

Agilent Xcalibur Gemini ultra diffractometer with Atlas detector	7053 independent reflections
Radiation source: Enhance Ultra (Cu) X-ray Source	5315 reflections with *I* > 3σ(*I*)
Mirror monochromator	*R*_int_ = 0.027
Detector resolution: 10.3784 pixels mm^-1^	θ_max_ = 67.1°, θ_min_ = 4.1°
ω scans	*h* = −24→28
Absorption correction: multi-scan (*CrysAlis PRO*; Agilent, 2010)	*k* = −11→10
*T*_min_ = 0.505, *T*_max_ = 1.000	*l* = −41→40
15999 measured reflections	

### Refinement

**Table:**

Refinement on *F*	231 constraints
*R*[*F*^2^ > 2σ(*F*^2^)] = 0.040	H atoms treated by a mixture of independent and constrained refinement
*wR*(*F*^2^) = 0.048	Weighting scheme based on measured s.u.'s *w* = 1/(σ^2^(*F*) + 0.0001*F*^2^)
*S* = 1.68	(Δ/σ)_max_ = 0.022
7053 reflections	Δρ_max_ = 0.31 e Å^−^^3^
556 parameters	Δρ_min_ = −0.19 e Å^−^^3^
7 restraints	

### Special details

**Table:**

Experimental. CrysAlisPro, Agilent, 2010 Empirical absorption correction using spherical harmonics, implemented in SCALE3 ABSPACK scaling algorithm.
Refinement. The refinement was carried out against all reflections. The conventional *R*-factor is always based on *F*. The goodness of fit as well as the weighted *R*-factor are based on *F* and *F*^2^ for refinement carried out on *F* and *F*^2^, respectively. The threshold expression is used only for calculating *R*-factors *etc*. and it is not relevant to the choice of reflections for refinement. The program used for refinement, Jana2006, uses the weighting scheme based on the experimental expectations, see _refine_ls_weighting_details, that does not force *S* to be one. Therefore the values of *S* are usually larger than the ones from the *SHELX* program.

### Fractional atomic coordinates and isotropic or equivalent isotropic displacement parameters (Å^2^)

**Table:**

	*x*	*y*	*z*	*U*_iso_*/*U*_eq_	Occ. (<1)
O1	0.95371 (6)	0.19541 (14)	0.01694 (4)	0.0419 (5)	
O2	0.78858 (5)	0.22409 (14)	0.12403 (4)	0.0378 (4)	
O3	0.78818 (5)	0.48488 (14)	0.19668 (4)	0.0387 (5)	
O4	0.94590 (5)	−0.01395 (14)	−0.13241 (3)	0.0364 (4)	
O5	0.95161 (5)	−0.02816 (13)	−0.04311 (3)	0.0325 (4)	
O6	0.94669 (6)	0.28240 (15)	0.34574 (4)	0.0432 (5)	
N2	0.87484 (6)	0.35663 (14)	0.24227 (4)	0.0265 (5)	
N3	0.86673 (6)	0.19986 (14)	−0.04268 (4)	0.0246 (4)	
N4	0.88014 (6)	0.26264 (14)	0.18386 (4)	0.0269 (5)	
N1	0.87030 (6)	0.12455 (14)	−0.10395 (4)	0.0270 (5)	
C45	0.92473 (8)	0.29225 (19)	0.03307 (5)	0.0320 (6)	
C46	0.82322 (7)	0.13953 (18)	0.10878 (5)	0.0295 (6)	
C47	0.95436 (9)	0.3752 (2)	0.06230 (5)	0.0418 (7)	
C48	0.88388 (7)	0.51481 (17)	0.18940 (4)	0.0270 (5)	
C1	0.90105 (7)	0.37983 (17)	0.20879 (5)	0.0264 (5)	
C2	0.85171 (7)	−0.04658 (18)	−0.05479 (5)	0.0278 (5)	
C3	0.92609 (10)	0.4723 (2)	0.07911 (5)	0.0424 (7)	
C4	0.85081 (10)	0.75819 (19)	0.15069 (5)	0.0413 (7)	
C5	0.79881 (8)	0.05083 (19)	0.07899 (5)	0.0346 (6)	
C6	0.86364 (11)	−0.3038 (2)	−0.01921 (6)	0.0477 (8)	
C7	0.82775 (8)	0.55781 (18)	0.18302 (5)	0.0312 (6)	
C8	0.83500 (7)	0.21722 (18)	−0.01195 (5)	0.0288 (6)	
C9	0.80471 (9)	−0.1247 (2)	−0.05198 (5)	0.0383 (7)	
C10	0.84272 (7)	0.09546 (17)	−0.07138 (5)	0.0267 (5)	
C11	0.87636 (11)	0.1786 (2)	−0.23652 (5)	0.0477 (8)	
C12	0.89522 (8)	0.34332 (18)	0.34300 (5)	0.0312 (6)	
C13	0.86650 (8)	0.30750 (17)	0.02005 (5)	0.0277 (5)	
C14	0.90529 (8)	−0.10177 (17)	−0.03976 (5)	0.0279 (5)	
C15	0.91066 (10)	−0.22970 (19)	−0.02197 (5)	0.0384 (7)	
C16	0.81141 (9)	0.67841 (19)	0.16319 (5)	0.0384 (7)	
C17	0.88192 (7)	0.14208 (17)	0.12213 (5)	0.0267 (5)	
C18	0.86977 (8)	0.20647 (18)	0.24694 (5)	0.0339 (6)	
C19	0.86484 (9)	0.3312 (2)	0.37219 (5)	0.0368 (6)	
C20	0.84506 (10)	0.1634 (2)	−0.20836 (5)	0.0438 (7)	
C21	0.83213 (8)	−0.0343 (2)	0.06214 (5)	0.0360 (6)	
C22	0.87397 (8)	0.41967 (18)	0.30986 (5)	0.0290 (6)	
C23	0.79147 (9)	0.47089 (19)	0.33567 (5)	0.0384 (7)	
C24	0.88997 (8)	−0.03312 (19)	0.07506 (5)	0.0349 (6)	
C25	0.91415 (8)	0.05456 (18)	0.10489 (5)	0.0313 (6)	
C26	0.86595 (14)	0.3233 (9)	−0.06671 (12)	0.0283 (8)	0.7544
C26'	0.8776 (5)	0.323 (3)	−0.0652 (4)	0.0283 (8)	0.2456
C27	0.88837 (8)	0.14813 (18)	0.21160 (5)	0.0324 (6)	
C28	0.90970 (7)	0.24526 (18)	0.15184 (5)	0.0297 (6)	
C29	0.92237 (8)	0.05111 (18)	−0.16700 (5)	0.0303 (6)	
C30	0.92320 (9)	0.59829 (18)	0.17732 (5)	0.0335 (6)	
C31	0.82189 (8)	0.48319 (19)	0.30694 (5)	0.0348 (6)	
C32	0.86847 (9)	0.4870 (2)	0.06741 (5)	0.0404 (7)	
C33	0.81058 (10)	−0.2528 (2)	−0.03426 (6)	0.0496 (8)	
C34	0.86738 (8)	0.09890 (19)	−0.17311 (5)	0.0313 (6)	
C35	0.90488 (8)	0.43120 (19)	0.27722 (5)	0.0316 (6)	
C36	0.83919 (9)	0.40474 (18)	0.03783 (5)	0.0352 (6)	
C37	0.81342 (9)	0.39430 (19)	0.36837 (5)	0.0382 (7)	
C38	0.95412 (9)	0.0660 (2)	−0.19492 (6)	0.0419 (7)	
C39	0.90683 (10)	0.71999 (19)	0.15794 (5)	0.0412 (7)	
C40	0.88940 (11)	0.2730 (3)	−0.10092 (7)	0.0297 (8)	0.7544
C40'	0.8611 (3)	0.2702 (10)	−0.1067 (3)	0.0297 (8)	0.2456
C41	0.93073 (10)	0.1306 (2)	−0.22976 (6)	0.0474 (8)	
C42	0.83485 (8)	0.0823 (2)	−0.14163 (5)	0.0390 (7)	
C43	0.80854 (9)	0.1690 (2)	0.24714 (6)	0.0444 (7)	
C44	0.95226 (11)	0.2901 (3)	−0.09486 (8)	0.0457 (10)	0.7544
H1C47	0.994508	0.365094	0.070848	0.0502*	
H1C1	0.941523	0.385416	0.21539	0.0317*	
H1C3	0.946827	0.530325	0.09917	0.0508*	
H1C4	0.839141	0.841134	0.136801	0.0495*	
H1C5	0.758507	0.048774	0.070097	0.0416*	
H1C6	0.867804	−0.391325	−0.006743	0.0572*	
H1C8	0.828765	0.129791	−0.001377	0.0345*	
H2C8	0.798809	0.256682	−0.022827	0.0345*	
H1C9	0.767564	−0.089786	−0.062453	0.046*	
H1C10	0.80234	0.09867	−0.079694	0.0321*	
H1C11	0.860151	0.22247	−0.260745	0.0572*	
H1C15	0.94756	−0.266204	−0.011581	0.0461*	
H1C16	0.772569	0.706364	0.158165	0.0461*	
H1C18	0.892046	0.171781	0.270903	0.0407*	
H1C19	0.879891	0.278525	0.395072	0.0442*	
H1C20	0.80716	0.197972	−0.213141	0.0525*	
H1C21	0.814971	−0.094283	0.041399	0.0432*	
H1C23	0.755492	0.514983	0.332976	0.0461*	
H1C24	0.913261	−0.092445	0.063519	0.0419*	
H1C25	0.954443	0.054698	0.113879	0.0375*	
H1C27	0.927746	0.125176	0.218571	0.0389*	
H2C27	0.86383	0.074385	0.200981	0.0389*	
H1C28	0.948094	0.218689	0.162231	0.0357*	
H2C28	0.911812	0.331383	0.139295	0.0357*	
H1C30	0.962178	0.571479	0.182448	0.0402*	
H1C31	0.806747	0.537041	0.284312	0.0418*	
H1C32	0.848835	0.553251	0.07959	0.0485*	
H1C33	0.777712	−0.305035	−0.032587	0.0595*	
H1C35	0.908257	0.525479	0.270808	0.0379*	
H2C35	0.942308	0.394912	0.285534	0.0379*	
H1C36	0.799047	0.415411	0.029464	0.0422*	
H1C37	0.792598	0.385145	0.388555	0.0458*	
H1C38	0.992093	0.031886	−0.190284	0.0503*	
H1C39	0.934258	0.77688	0.149677	0.0494*	
H1C41	0.952701	0.141814	−0.249219	0.0568*	
H1C42	0.801503	0.137883	−0.147465	0.0468*	
H2C42	0.824181	−0.011411	−0.14019	0.0468*	
H1C43	0.805315	0.071928	0.249169	0.0532*	
H2C43	0.79764	0.211737	0.268958	0.0532*	
H3C43	0.784286	0.199906	0.223437	0.0532*	
H1C44	0.964516	0.259305	−0.117596	0.0548*	0.7544
H2C44	0.961823	0.384565	−0.090395	0.0548*	0.7544
H3C44	0.970606	0.237512	−0.072721	0.0548*	0.7544
H5	0.9826 (6)	−0.0767 (17)	−0.0353 (5)	0.039*	
H1	0.9318 (8)	0.172 (2)	−0.0056 (4)	0.0503*	
H2	0.8099 (8)	0.260 (2)	0.1452 (4)	0.0454*	
H3	0.8089 (8)	0.4317 (18)	0.2144 (5)	0.0464*	
H4	0.9296 (8)	0.0246 (19)	−0.1150 (5)	0.0437*	
H6	0.9458 (9)	0.2057 (14)	0.3587 (6)	0.0519*	
C44'	0.9383 (3)	0.3680 (9)	−0.0554 (2)	0.046 (3)	0.2456
H1C26	0.891045	0.390171	−0.052417	0.0339*	0.7544
H2C26	0.827656	0.352997	−0.075848	0.0339*	0.7544
H1C40	0.876076	0.323585	−0.124471	0.0356*	0.7544
H1C44'	0.961796	0.295871	−0.061466	0.0556*	0.2456
H2C44'	0.942857	0.447344	−0.070407	0.0556*	0.2456
H3C44'	0.949117	0.389126	−0.02818	0.0556*	0.2456
H1C26'	0.857458	0.402884	−0.059874	0.0339*	0.2456
H1C40'	0.821774	0.288173	−0.11693	0.0356*	0.2456
H2C40'	0.885218	0.309841	−0.122001	0.0356*	0.2456

### Atomic displacement parameters (Å^2^)

**Table:**

	*U*^11^	*U*^22^	*U*^33^	*U*^12^	*U*^13^	*U*^23^
O1	0.0347 (8)	0.0531 (9)	0.0331 (7)	0.0104 (7)	−0.0034 (6)	−0.0128 (6)
O2	0.0272 (7)	0.0461 (8)	0.0412 (7)	0.0076 (6)	0.0094 (6)	0.0000 (6)
O3	0.0290 (7)	0.0408 (8)	0.0455 (8)	0.0031 (6)	0.0061 (6)	0.0078 (6)
O4	0.0315 (7)	0.0484 (8)	0.0300 (7)	0.0092 (6)	0.0080 (5)	0.0007 (6)
O5	0.0257 (7)	0.0311 (7)	0.0387 (7)	0.0019 (6)	0.0022 (5)	0.0043 (5)
O6	0.0355 (8)	0.0516 (9)	0.0413 (8)	0.0016 (7)	0.0055 (6)	0.0141 (6)
N2	0.0295 (8)	0.0268 (8)	0.0248 (7)	−0.0020 (6)	0.0092 (6)	0.0023 (6)
N3	0.0265 (8)	0.0252 (7)	0.0231 (7)	−0.0003 (6)	0.0078 (6)	0.0002 (5)
N4	0.0295 (8)	0.0246 (7)	0.0288 (7)	0.0011 (6)	0.0111 (6)	0.0044 (6)
N1	0.0283 (8)	0.0307 (8)	0.0214 (7)	−0.0002 (7)	0.0040 (6)	−0.0004 (6)
C45	0.0384 (11)	0.0342 (10)	0.0245 (8)	−0.0008 (9)	0.0090 (7)	−0.0015 (7)
C46	0.0301 (10)	0.0314 (9)	0.0290 (9)	0.0030 (8)	0.0111 (7)	0.0083 (7)
C47	0.0438 (12)	0.0495 (12)	0.0319 (10)	−0.0102 (10)	0.0074 (9)	−0.0068 (9)
C48	0.0333 (10)	0.0249 (9)	0.0233 (8)	−0.0007 (8)	0.0073 (7)	0.0004 (6)
C1	0.0244 (9)	0.0292 (9)	0.0268 (8)	−0.0002 (8)	0.0081 (7)	0.0026 (7)
C2	0.0312 (10)	0.0306 (9)	0.0237 (8)	−0.0072 (8)	0.0102 (7)	−0.0064 (7)
C3	0.0607 (14)	0.0411 (11)	0.0287 (9)	−0.0196 (11)	0.0173 (9)	−0.0084 (8)
C4	0.0696 (15)	0.0242 (10)	0.0299 (10)	0.0028 (10)	0.0102 (9)	0.0040 (7)
C5	0.0285 (10)	0.0416 (11)	0.0329 (9)	−0.0037 (9)	0.0046 (8)	0.0072 (8)
C6	0.0780 (17)	0.0314 (11)	0.0403 (11)	−0.0098 (12)	0.0275 (11)	−0.0033 (8)
C7	0.0351 (10)	0.0299 (9)	0.0279 (9)	−0.0018 (8)	0.0056 (7)	0.0003 (7)
C8	0.0290 (9)	0.0317 (9)	0.0274 (8)	−0.0004 (8)	0.0102 (7)	0.0002 (7)
C9	0.0379 (11)	0.0419 (11)	0.0397 (10)	−0.0140 (9)	0.0184 (9)	−0.0160 (8)
C10	0.0202 (9)	0.0338 (10)	0.0260 (8)	−0.0021 (8)	0.0047 (7)	−0.0033 (7)
C11	0.0825 (18)	0.0329 (11)	0.0263 (10)	−0.0001 (11)	0.0089 (10)	0.0015 (8)
C12	0.0304 (10)	0.0323 (10)	0.0297 (9)	−0.0032 (8)	0.0041 (7)	−0.0005 (7)
C13	0.0352 (10)	0.0272 (9)	0.0230 (8)	−0.0023 (8)	0.0111 (7)	0.0025 (7)
C14	0.0345 (10)	0.0276 (9)	0.0219 (8)	−0.0048 (8)	0.0071 (7)	−0.0034 (7)
C15	0.0586 (14)	0.0296 (10)	0.0278 (9)	−0.0006 (10)	0.0109 (9)	−0.0007 (7)
C16	0.0466 (12)	0.0350 (11)	0.0311 (9)	0.0101 (10)	0.0026 (9)	0.0021 (8)
C17	0.0268 (9)	0.0286 (9)	0.0263 (8)	0.0009 (8)	0.0095 (7)	0.0069 (7)
C18	0.0430 (11)	0.0288 (9)	0.0326 (9)	0.0040 (9)	0.0140 (8)	0.0068 (7)
C19	0.0485 (12)	0.0352 (10)	0.0260 (9)	−0.0087 (9)	0.0063 (8)	0.0026 (8)
C20	0.0532 (13)	0.0447 (12)	0.0296 (10)	0.0138 (10)	0.0004 (9)	−0.0035 (8)
C21	0.0423 (12)	0.0368 (10)	0.0288 (9)	−0.0073 (9)	0.0073 (8)	0.0017 (8)
C22	0.0331 (10)	0.0286 (9)	0.0257 (8)	−0.0065 (8)	0.0074 (7)	−0.0020 (7)
C23	0.0408 (12)	0.0339 (10)	0.0445 (11)	−0.0013 (9)	0.0179 (9)	−0.0031 (8)
C24	0.0419 (11)	0.0328 (10)	0.0331 (9)	0.0023 (9)	0.0149 (8)	0.0010 (8)
C25	0.0284 (10)	0.0352 (10)	0.0317 (9)	0.0024 (8)	0.0100 (8)	0.0046 (7)
C26	0.0286 (17)	0.0292 (10)	0.0274 (9)	−0.001 (2)	0.0069 (11)	0.0027 (8)
C26'	0.0286 (17)	0.0292 (10)	0.0274 (9)	−0.001 (2)	0.0069 (11)	0.0027 (8)
C27	0.0372 (11)	0.0279 (9)	0.0335 (9)	0.0035 (8)	0.0106 (8)	0.0063 (7)
C28	0.0270 (9)	0.0335 (10)	0.0317 (9)	0.0008 (8)	0.0130 (7)	0.0029 (7)
C29	0.0321 (10)	0.0313 (9)	0.0276 (9)	−0.0044 (8)	0.0069 (7)	−0.0031 (7)
C30	0.0405 (11)	0.0320 (10)	0.0294 (9)	−0.0052 (9)	0.0105 (8)	−0.0012 (7)
C31	0.0381 (11)	0.0324 (10)	0.0352 (10)	0.0004 (9)	0.0108 (8)	0.0022 (8)
C32	0.0614 (14)	0.0312 (10)	0.0349 (10)	−0.0032 (10)	0.0244 (9)	−0.0045 (8)
C33	0.0659 (16)	0.0401 (12)	0.0533 (12)	−0.0272 (12)	0.0364 (11)	−0.0151 (10)
C34	0.0322 (10)	0.0358 (10)	0.0251 (9)	0.0022 (8)	0.0042 (7)	−0.0052 (7)
C35	0.0293 (10)	0.0370 (10)	0.0280 (9)	−0.0033 (8)	0.0052 (7)	0.0017 (7)
C36	0.0436 (12)	0.0351 (10)	0.0303 (9)	−0.0011 (9)	0.0156 (8)	0.0012 (8)
C37	0.0496 (12)	0.0357 (10)	0.0343 (10)	−0.0097 (10)	0.0202 (9)	−0.0080 (8)
C38	0.0379 (11)	0.0512 (12)	0.0401 (11)	−0.0100 (10)	0.0161 (9)	−0.0080 (9)
C39	0.0628 (15)	0.0291 (10)	0.0348 (10)	−0.0097 (10)	0.0175 (9)	0.0019 (8)
C40	0.0323 (16)	0.0304 (11)	0.0286 (12)	−0.0050 (15)	0.0117 (14)	−0.0006 (9)
C40'	0.0323 (16)	0.0304 (11)	0.0286 (12)	−0.0050 (15)	0.0117 (14)	−0.0006 (9)
C41	0.0679 (16)	0.0454 (12)	0.0342 (11)	−0.0190 (12)	0.0229 (10)	−0.0054 (9)
C42	0.0252 (10)	0.0641 (14)	0.0255 (9)	0.0040 (10)	0.0006 (7)	−0.0089 (9)
C43	0.0505 (13)	0.0336 (11)	0.0552 (12)	−0.0045 (10)	0.0251 (10)	0.0014 (9)
C44	0.0408 (15)	0.0439 (16)	0.0599 (18)	−0.0174 (13)	0.0277 (13)	−0.0201 (13)
C44'	0.046 (5)	0.058 (6)	0.032 (4)	−0.020 (5)	0.003 (4)	0.007 (4)

### Geometric parameters (Å, º)

**Table:**

O1—C45	1.375 (2)	C18—H1C18	0.96
O1—H1	0.883 (14)	C19—C37	1.372 (3)
O2—C46	1.370 (2)	C19—H1C19	0.96
O2—H2	0.883 (15)	C20—C34	1.393 (2)
O3—C7	1.365 (2)	C20—H1C20	0.96
O3—H3	0.883 (16)	C21—C24	1.378 (3)
O4—C29	1.383 (2)	C21—H1C21	0.96
O4—H4	0.883 (19)	C22—C31	1.392 (3)
O5—C14	1.362 (2)	C22—C35	1.504 (3)
O5—H5	0.883 (14)	C23—C31	1.379 (3)
O6—C12	1.369 (2)	C23—C37	1.381 (3)
O6—H6	0.883 (16)	C23—H1C23	0.96
N2—C1	1.471 (2)	C24—C25	1.385 (2)
N2—C18	1.491 (2)	C24—H1C24	0.96
N2—C35	1.480 (2)	C25—H1C25	0.96
N3—C8	1.466 (2)	C26—C26'	0.277 (11)
N3—C10	1.468 (2)	C26—C40	1.520 (6)
N3—C26	1.475 (7)	C26—C40'	1.479 (10)
N3—C26'	1.50 (2)	C26—H1C26	0.96
N4—C1	1.469 (2)	C26—H2C26	0.96
N4—C27	1.473 (2)	C26—H1C26'	0.8552
N4—C28	1.469 (2)	C26'—C40	1.436 (16)
N1—C10	1.473 (2)	C26'—C40'	1.520 (17)
N1—C40	1.525 (3)	C26'—C44'	1.502 (15)
N1—C40'	1.447 (10)	C26'—H1C26	0.8205
N1—C42	1.475 (2)	C26'—H1C26'	0.96
C45—C47	1.384 (2)	C27—H1C27	0.96
C45—C13	1.395 (3)	C27—H2C27	0.96
C46—C5	1.392 (2)	C28—H1C28	0.96
C46—C17	1.400 (2)	C28—H2C28	0.96
C47—C3	1.380 (3)	C29—C34	1.386 (3)
C47—H1C47	0.96	C29—C38	1.380 (3)
C48—C1	1.507 (2)	C30—C39	1.390 (3)
C48—C7	1.396 (3)	C30—H1C30	0.96
C48—C30	1.391 (3)	C31—H1C31	0.96
C1—H1C1	0.96	C32—C36	1.385 (2)
C2—C9	1.395 (3)	C32—H1C32	0.96
C2—C10	1.509 (2)	C33—H1C33	0.96
C2—C14	1.402 (2)	C34—C42	1.500 (3)
C3—C32	1.376 (3)	C35—H1C35	0.96
C3—H1C3	0.96	C35—H2C35	0.96
C4—C16	1.379 (3)	C36—H1C36	0.96
C4—C39	1.379 (3)	C37—H1C37	0.96
C4—H1C4	0.96	C38—C41	1.388 (3)
C5—C21	1.381 (3)	C38—H1C38	0.96
C5—H1C5	0.96	C39—H1C39	0.96
C6—C15	1.373 (3)	C40—C40'	0.673 (8)
C6—C33	1.376 (3)	C40—C44	1.502 (4)
C6—H1C6	0.96	C40—H1C40	0.96
C7—C16	1.388 (2)	C40—H2C40'	0.8114
C8—C13	1.505 (2)	C40'—H1C40	0.9475
C8—H1C8	0.96	C40'—H1C40'	0.96
C8—H2C8	0.96	C40'—H2C40'	0.96
C9—C33	1.396 (3)	C41—H1C41	0.96
C9—H1C9	0.96	C42—H1C42	0.96
C10—H1C10	0.96	C42—H2C42	0.96
C11—C20	1.379 (3)	C43—H1C43	0.96
C11—C41	1.371 (4)	C43—H2C43	0.96
C11—H1C11	0.96	C43—H3C43	0.96
C12—C19	1.392 (3)	C44—H1C44	0.96
C12—C22	1.388 (2)	C44—H2C44	0.96
C13—C36	1.385 (3)	C44—H3C44	0.96
C14—C15	1.396 (2)	H3C44—H1C44'	0.7529
C15—H1C15	0.96	C44'—H1C44'	0.96
C16—H1C16	0.96	C44'—H2C44'	0.96
C17—C25	1.386 (3)	C44'—H3C44'	0.96
C17—C28	1.506 (2)	H1C26—H1C26'	0.8097
C18—C27	1.522 (3)	H1C40—H2C40'	0.258
C18—C43	1.531 (3)		
			
C45—O1—H1	106.7 (13)	N3—C26'—H1C26	116.81
C46—O2—H2	106.0 (13)	N3—C26'—H1C26'	113.51
C7—O3—H3	102.9 (13)	C26—C26'—C40	102 (3)
C29—O4—H4	104.5 (11)	C26—C26'—C40'	76 (3)
C14—O5—H5	110.5 (11)	C26—C26'—C44'	163 (7)
C12—O6—H6	106.6 (15)	C26—C26'—H1C26	112.21
C1—N2—C18	107.53 (13)	C26—C26'—H1C26'	59.85
C1—N2—C35	111.58 (13)	C40—C26'—C40'	26.1 (4)
C18—N2—C35	115.78 (12)	C40—C26'—C44'	86.0 (7)
C8—N3—C10	113.25 (13)	C40—C26'—H1C26	128.59
C8—N3—C26	112.5 (2)	C40—C26'—H1C26'	130.97
C8—N3—C26'	118.3 (7)	C40'—C26'—C44'	111.2 (9)
C10—N3—C26	103.0 (2)	C40'—C26'—H1C26	142.25
C10—N3—C26'	106.9 (7)	C40'—C26'—H1C26'	115.17
C26—N3—C26'	10.6 (4)	C44'—C26'—H1C26	52.38
C1—N4—C27	102.83 (12)	C44'—C26'—H1C26'	103.56
C1—N4—C28	112.68 (14)	H1C26—C26'—H1C26'	53.4
C27—N4—C28	113.58 (14)	N4—C27—C18	103.41 (14)
C10—N1—C40	108.22 (15)	N4—C27—H1C27	109.47
C10—N1—C40'	98.8 (4)	N4—C27—H2C27	109.47
C10—N1—C42	111.70 (13)	C18—C27—H1C27	109.47
C40—N1—C40'	26.0 (3)	C18—C27—H2C27	109.47
C40—N1—C42	116.13 (15)	H1C27—C27—H2C27	114.92
C40'—N1—C42	99.6 (3)	N4—C28—C17	113.00 (14)
O1—C45—C47	118.88 (17)	N4—C28—H1C28	109.47
O1—C45—C13	120.66 (15)	N4—C28—H2C28	109.47
C47—C45—C13	120.46 (18)	C17—C28—H1C28	109.47
O2—C46—C5	118.46 (15)	C17—C28—H2C28	109.47
O2—C46—C17	121.52 (14)	H1C28—C28—H2C28	105.7
C5—C46—C17	120.01 (17)	O4—C29—C34	119.56 (16)
C45—C47—C3	119.88 (19)	O4—C29—C38	119.14 (16)
C45—C47—H1C47	120.06	C34—C29—C38	121.30 (16)
C3—C47—H1C47	120.06	C48—C30—C39	120.92 (19)
C1—C48—C7	120.21 (16)	C48—C30—H1C30	119.54
C1—C48—C30	121.01 (16)	C39—C30—H1C30	119.54
C7—C48—C30	118.79 (16)	C22—C31—C23	121.81 (16)
N2—C1—N4	101.73 (13)	C22—C31—H1C31	119.1
N2—C1—C48	111.92 (14)	C23—C31—H1C31	119.1
N2—C1—H1C1	114.25	C3—C32—C36	119.13 (19)
N4—C1—C48	113.09 (12)	C3—C32—H1C32	120.43
N4—C1—H1C1	113.11	C36—C32—H1C32	120.44
C48—C1—H1C1	103.16	C6—C33—C9	119.7 (2)
C9—C2—C10	118.91 (15)	C6—C33—H1C33	120.13
C9—C2—C14	117.77 (16)	C9—C33—H1C33	120.13
C10—C2—C14	123.21 (16)	C20—C34—C29	117.91 (18)
C47—C3—C32	120.67 (18)	C20—C34—C42	122.73 (17)
C47—C3—H1C3	119.66	C29—C34—C42	119.35 (15)
C32—C3—H1C3	119.66	N2—C35—C22	111.06 (14)
C16—C4—C39	120.63 (18)	N2—C35—H1C35	109.47
C16—C4—H1C4	119.69	N2—C35—H2C35	109.47
C39—C4—H1C4	119.69	C22—C35—H1C35	109.47
C46—C5—C21	120.52 (17)	C22—C35—H2C35	109.47
C46—C5—H1C5	119.74	H1C35—C35—H2C35	107.83
C21—C5—H1C5	119.74	C13—C36—C32	121.52 (19)
C15—C6—C33	120.14 (19)	C13—C36—H1C36	119.24
C15—C6—H1C6	119.93	C32—C36—H1C36	119.24
C33—C6—H1C6	119.93	C19—C37—C23	120.4 (2)
O3—C7—C48	121.33 (15)	C19—C37—H1C37	119.8
O3—C7—C16	118.56 (17)	C23—C37—H1C37	119.8
C48—C7—C16	120.09 (18)	C29—C38—C41	119.5 (2)
N3—C8—C13	110.95 (14)	C29—C38—H1C38	120.26
N3—C8—H1C8	109.47	C41—C38—H1C38	120.26
N3—C8—H2C8	109.47	C4—C39—C30	119.3 (2)
C13—C8—H1C8	109.47	C4—C39—H1C39	120.33
C13—C8—H2C8	109.47	C30—C39—H1C39	120.33
H1C8—C8—H2C8	107.95	N1—C40—C26	101.9 (3)
C2—C9—C33	121.36 (18)	N1—C40—C26'	106.0 (10)
C2—C9—H1C9	119.32	N1—C40—C40'	70.4 (9)
C33—C9—H1C9	119.32	N1—C40—C44	113.6 (2)
N3—C10—N1	102.71 (13)	N1—C40—H1C40	113.15
N3—C10—C2	112.10 (12)	N1—C40—H2C40'	112.69
N3—C10—H1C10	114.45	C26—C40—C26'	10.2 (4)
N1—C10—C2	115.33 (15)	C26—C40—C40'	73.7 (8)
N1—C10—H1C10	111.16	C26—C40—C44	113.1 (2)
C2—C10—H1C10	101.59	C26—C40—H1C40	113.65
C20—C11—C41	119.70 (18)	C26—C40—H2C40'	125.74
C20—C11—H1C11	120.15	C26'—C40—C40'	83.9 (9)
C41—C11—H1C11	120.15	C26'—C40—C44	103.0 (5)
O6—C12—C19	122.05 (15)	C26'—C40—H1C40	118.92
O6—C12—C22	117.56 (17)	C26'—C40—H2C40'	129.57
C19—C12—C22	120.39 (17)	C40'—C40—C44	170.1 (9)
C45—C13—C8	119.78 (16)	C40'—C40—H1C40	68.34
C45—C13—C36	118.31 (15)	C40'—C40—H2C40'	79.99
C8—C13—C36	121.89 (16)	C44—C40—H1C40	101.93
O5—C14—C2	118.51 (15)	C44—C40—H2C40'	90.15
O5—C14—C15	121.11 (16)	H1C40—C40—H2C40'	13.73
C2—C14—C15	120.38 (18)	N1—C40'—C26	107.8 (6)
C6—C15—C14	120.60 (19)	N1—C40'—C26'	105.8 (11)
C6—C15—H1C15	119.7	N1—C40'—C40	83.5 (9)
C14—C15—H1C15	119.7	N1—C40'—H1C40	120.94
C4—C16—C7	120.16 (19)	N1—C40'—H1C40'	109.47
C4—C16—H1C16	119.92	N1—C40'—H2C40'	109.47
C7—C16—H1C16	119.92	C26—C40'—C26'	10.5 (5)
C46—C17—C25	118.08 (15)	C26—C40'—C40	80.4 (8)
C46—C17—C28	121.01 (16)	C26—C40'—H1C40	118
C25—C17—C28	120.69 (15)	C26—C40'—H1C40'	99.32
N2—C18—C27	103.64 (15)	C26—C40'—H2C40'	117.22
N2—C18—C43	109.93 (15)	C26'—C40'—C40	69.9 (8)
N2—C18—H1C18	113.77	C26'—C40'—H1C40	112.64
C27—C18—C43	111.83 (15)	C26'—C40'—H1C40'	109.47
C27—C18—H1C18	111.94	C26'—C40'—H2C40'	109.47
C43—C18—H1C18	105.87	C40—C40'—H1C40	70.33
C12—C19—C37	120.23 (16)	C40—C40'—H1C40'	166.15
C12—C19—H1C19	119.89	C40—C40'—H2C40'	56.34
C37—C19—H1C19	119.89	H1C40—C40'—H1C40'	98.09
C11—C20—C34	121.3 (2)	H1C40—C40'—H2C40'	15.53
C11—C20—H1C20	119.35	H1C40'—C40'—H2C40'	112.95
C34—C20—H1C20	119.35	C11—C41—C38	120.3 (2)
C5—C21—C24	120.11 (16)	C11—C41—H1C41	119.85
C5—C21—H1C21	119.95	C38—C41—H1C41	119.84
C24—C21—H1C21	119.95	N1—C42—C34	109.58 (15)
C12—C22—C31	118.04 (17)	N1—C42—H1C42	109.47
C12—C22—C35	121.71 (16)	N1—C42—H2C42	109.47
C31—C22—C35	120.23 (15)	C34—C42—H1C42	109.47
C31—C23—C37	119.13 (19)	C34—C42—H2C42	109.47
C31—C23—H1C23	120.44	H1C42—C42—H2C42	109.36
C37—C23—H1C23	120.44	C18—C43—H1C43	109.47
C21—C24—C25	119.29 (18)	C18—C43—H2C43	109.47
C21—C24—H1C24	120.36	C18—C43—H3C43	109.47
C25—C24—H1C24	120.36	H1C43—C43—H2C43	109.47
C17—C25—C24	121.99 (17)	H1C43—C43—H3C43	109.47
C17—C25—H1C25	119	H2C43—C43—H3C43	109.47
C24—C25—H1C25	119	C40—C44—H1C44	109.47
N3—C26—C26'	90 (5)	C40—C44—H2C44	109.47
N3—C26—C40	103.0 (5)	C40—C44—H3C44	109.47
N3—C26—C40'	104.1 (6)	H1C44—C44—H2C44	109.47
N3—C26—H1C26	109.47	H1C44—C44—H3C44	109.47
N3—C26—H2C26	109.47	H2C44—C44—H3C44	109.47
N3—C26—H1C26'	124.3	C44—H3C44—H1C44'	83.26
C26'—C26—C40	67 (3)	C26'—C44'—H1C44'	109.47
C26'—C26—C40'	93 (3)	C26'—C44'—H2C44'	109.47
C26'—C26—H1C26	52.3	C26'—C44'—H3C44'	109.47
C26'—C26—H2C26	160.01	H1C44'—C44'—H2C44'	109.47
C26'—C26—H1C26'	103.89	H1C44'—C44'—H3C44'	109.47
C40—C26—C40'	25.9 (3)	H2C44'—C44'—H3C44'	109.47
C40—C26—H1C26	109.47	C26—H1C26—C26'	15.48
C40—C26—H2C26	109.47	C26—H1C26—H1C26'	57.04
C40—C26—H1C26'	132.3	C26'—H1C26—H1C26'	72.15
C40'—C26—H1C26	130.53	C40—H1C40—C40'	41.33
C40'—C26—H2C26	85.15	C40—H1C40—H2C40'	48.26
C40'—C26—H1C26'	127.88	C40'—H1C40—H2C40'	84.98
H1C26—C26—H2C26	115.22	H3C44—H1C44'—C44'	157.81
H1C26—C26—H1C26'	52.59	C26—H1C26'—C26'	16.26
H2C26—C26—H1C26'	62.8	C26—H1C26'—H1C26	70.37
N3—C26'—C26	79 (5)	C26'—H1C26'—H1C26	54.45
N3—C26'—C40	105.9 (17)	C40—H2C40'—C40'	43.68
N3—C26'—C40'	100.8 (15)	C40—H2C40'—H1C40	118.01
N3—C26'—C44'	112.9 (11)	C40'—H2C40'—H1C40	79.49

### Hydrogen-bond geometry (Å, º)

**Table:**

*D*—H···*A*	*D*—H	H···*A*	*D*···*A*	*D*—H···*A*
O1—H1···N3	0.883 (14)	1.838 (16)	2.6344 (17)	148.9 (19)
O2—H2···N4	0.883 (15)	1.937 (16)	2.7360 (17)	149.7 (18)
O3—H3···N2	0.883 (16)	1.839 (17)	2.6680 (17)	155.5 (19)
O4—H4···N1	0.883 (19)	1.849 (19)	2.646 (2)	149.0 (14)
O1—H1···O5	0.883 (14)	2.472 (19)	3.0381 (18)	122.4 (16)
O4—H4···O5	0.883 (14)	2.523 (17)	3.1167 (15)	125.3 (15)
O5—H5···O1^i^	0.883 (14)	1.930 (15)	2.8108 (18)	174.9 (16)
O6—H6···O4^ii^	0.883 (16)	1.907 (14)	2.746 (2)	157.9 (18)
C26—H1*C*26···C44′	0.96	1.19	1.769 (9)	109.96
C26′—H1*C*26···C44′	0.82	1.19	1.502 (15)	94.63
C44′—H1*C*26···N3	1.19	2.01	2.504 (9)	99.67
C44′—H1*C*26···C26	1.19	0.96	1.769 (9)	109.96
C44′—H1*C*26···C26′	1.19	0.82	1.502 (15)	94.63
C40′—H1*C*40···C44	0.95	1.94	2.169 (9)	90.47
C44—H1*C*44′···C44′	1.15	0.96	1.680 (9)	105.31
C44′—H1*C*44′···C44	0.96	1.15	1.680 (9)	105.31
C44′—H3*C*44′···O1	0.96	2.46	3.012 (8)	116.14
C40′—H2*C*40′···C44	0.96	1.71	2.169 (9)	105.19
C25—H1*C*25···O4^i^	0.96	2.40	3.346 (2)	169.82
C35—H2*C*35···O6	0.96	2.37	2.813 (2)	107.61
O1—H1···C8	0.883 (14)	2.352 (19)	2.847 (2)	115.5 (15)
O2—H2···C28	0.883 (15)	2.385 (19)	2.896 (2)	117.1 (15)
O3—H3···C1	0.883 (16)	2.34 (2)	2.869 (2)	118.5 (16)
O4—H4···C42	0.883 (19)	2.355 (18)	2.806 (2)	111.8 (12)

Symmetry codes: (i) −*x*+2, −*y*, −*z*; (ii) *x*, −*y*, *z*+1/2.
